# The Epidemiology of Chronic Kidney Disease in Northern Tanzania: A Population-Based Survey

**DOI:** 10.1371/journal.pone.0124506

**Published:** 2015-04-17

**Authors:** John W. Stanifer, Venance Maro, Joseph Egger, Francis Karia, Nathan Thielman, Elizabeth L. Turner, Dionis Shimbi, Humphrey Kilaweh, Oliver Matemu, Uptal D. Patel

**Affiliations:** 1 Department of Medicine, Duke University, Durham, North Carolina, United States of America; 2 Duke Global Health Institute, Duke University, Durham, North Carolina, United States of America; 3 Kilimanjaro Christian Medical College, Moshi, Tanzania; 4 Department of Biostatistics and Bioinformatics, Duke University, Durham, North Carolina, United States of America; 5 Duke Clinical Research Institute, Duke University, Durham, North Carolina, United States of America; National Cancer Institute, UNITED STATES

## Abstract

**Background:**

In sub-Saharan Africa, kidney failure has a high morbidity and mortality. Despite this, population-based estimates of prevalence, potential etiologies, and awareness are not available.

**Methods:**

Between January and June 2014, we conducted a household survey of randomly-selected adults in Northern Tanzania. To estimate prevalence we screened for CKD, which was defined as an estimated glomerular filtration rate ≤ 60 ml/min/1.73m2 and/or persistent albuminuria. We also screened for human immunodeficiency virus (HIV), diabetes, hypertension, obesity, and lifestyle practices including alcohol, tobacco, and traditional medicine use. Awareness was defined as a self-reported disease history and subsequently testing positive. We used population-based age- and gender-weights in estimating prevalence, and we used generalized linear models to explore potential risk factors associated with CKD, including living in an urban environment.

**Results:**

We enrolled 481 adults from 346 households with a median age of 45 years. The community-based prevalence of CKD was 7.0% (95% CI 3.8-12.3), and awareness was low at 10.5% (4.7-22.0). The urban prevalence of CKD was 15.2% (9.6-23.3) while the rural prevalence was 2.0% (0.5-6.9). Half of the cases of CKD (49.1%) were not associated with any of the measured risk factors of hypertension, diabetes, or HIV. Living in an urban environment had the strongest crude (5.40; 95% CI 2.05-14.2) and adjusted prevalence risk ratio (4.80; 1.70-13.6) for CKD, and the majority (79%) of this increased risk was not explained by demographics, traditional medicine use, socioeconomic status, or co-morbid non-communicable diseases (NCDs).

**Conclusions:**

We observed a high burden of CKD in Northern Tanzania that was associated with low awareness. Although demographic, lifestyle practices including traditional medicine use, socioeconomic factors, and NCDs accounted for some of the excess CKD risk observed with urban residence, much of the increased urban prevalence remained unexplained and will further study as demographic shifts reshape sub-Saharan Africa.

## Introduction

Non-communicable diseases (NCDs) are a growing health burden that disproportionately affect the economic, social, and health outcomes of lower- and middle-income countries (LMIC) [1]. They are one of the most significant barriers to achieving the United Nation’s Millennium Development Goals and now account for 60% of all deaths worldwide [2, 3]. NCDs are largely driven by urbanization, and amid rapid urbanization and demographic transitions, sub-Saharan Africa may be particularly vulnerable [2–4]. In Tanzania, for example, the urban population has increased from less than 5% in 1970 to nearly 30% today [5].

As an NCD that is associated with both communicable and non-communicable risk factors, chronic kidney disease (CKD) has the potential to be especially burdensome in sub-Saharan Africa. Given the increased cardiovascular morbidity and mortality and the near uniform fatality of end-stage kidney disease in the region, early detection and recognition of CKD is essential [6]. However, despite this need, little is known about its prevalence and potential etiologies [7]. We previously identified four high-quality epidemiological studies from the entire sub-continent, and only two of these, both from Western Africa, explored urban and rural differences [7–11]. Thus, there are no community-based epidemiological data for CKD in East Africa even though there is a high burden of potential underlying risk factors such as hypertension, diabetes, and human immunodeficiency virus (HIV) [12–15].

The Comprehensive Kidney Disease Assessment for Risk Factors, epIdemiology, Knowledge, and Attitudes (CKD-AFRIKA) study is an ongoing project in Northern Tanzania with the goal of understanding and addressing the health burden of CKD. The specific aim of this study was to estimate the prevalence, evaluate potential etiologies, and examine awareness of CKD so that we can better inform public health efforts directed toward CKD.

## Methods

### Ethics Statement

The study protocol was approved by Duke University Institutional Review Board (#Pro00040784), the Kilimanjaro Christian Medical College (KCMC) Ethics Committee (EC#502), and the National Institute for Medical Research (NIMR) in Tanzania. Written informed consent (by signature or thumbprint) was obtained from all participants, and all participants with abnormal findings received counseling, educational pamphlets, and reimbursement with referral for follow-up.

### Study Setting and Design

**Fig 1:**
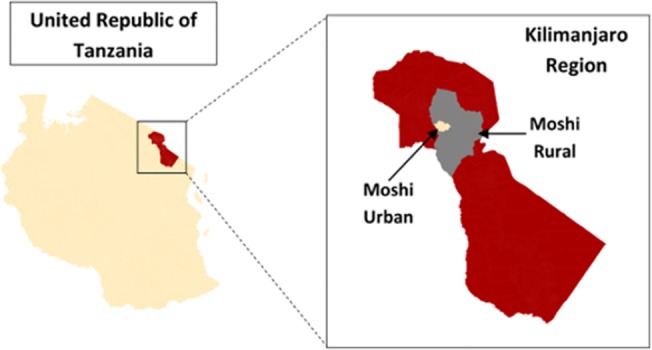
Study Setting. Map showing the sampling area of Moshi Urban and Moshi Rural in the Kilimanjaro Region of Northern Tanzania.

We conducted a stratified, cluster-designed cross-sectional survey between January and June 2014 in the Kilimanjaro Region of Tanzania. The adult regional population is more than 900,000 people, and it has a female majority (58%). Almost 35% of the adult population lives in an urban setting, which is comparable to national estimates, and the HIV prevalence is 3–5% which is slightly lower than the national prevalence of 5–6%. The unemployment rate is 19%, and most people have only a primary education (77%); however, the region is slightly more educated with literacy rates of 80% compared to the national average of 72%. The median age, average household size, and occupation distribution are no different from national estimates. The largest ethnic group is the Chagga tribe followed by the Pare, Sambaa, and Maasai tribes, and Swahili is the major language. The region comprises seven districts; our study was conducted in the Moshi Urban and Moshi Rural districts (**Fig 1**) [5, 16, 17].

We used a three-stage cluster probability sampling method stratified by urban and rural. Using a random-number generator, we chose thirty-seven neighborhoods (from a total of 230) from the Moshi Urban and Moshi Rural districts based on probability proportional to size according to the 2012 national census [5]. Neighborhoods (also known as streets) are the most basic governmental administrative unit in Tanzania and range in size from 500 to 3000 people. Within each selected neighborhood a cluster site was determined using geographic points randomly generated by Arc Global Information Systems (ArcGIS), v10.2.2 (Environmental Systems Research Institute, Redlands, CA). From the cluster site, households were then randomly chosen based on both a coin-flip and die-rolling technique according to our established protocol (**S1 Appendix**). To ensure that the protocol was strictly adhered to, the principal investigator (JWS) regularly accompanied the surveyors and frequently conducted anonymous shadowing.

All non-pregnant adults (age ≥ 18 years old) living in the selected households were recruited. To be eligible each adult had to be a permanent resident (living ≥ nine months each year in Tanzania) or a citizen of Tanzania. The sample size was developed to estimate the prevalence of CKD with a precision of 5% when accounting for the design effect, and we targeted 10 to 15 participants per cluster. The household non-response rate was 15.0% and the individual non-response rate was 20.6%. To reduce non-response rates, we attempted a minimum of two additional visits during off-hours (evenings and weekends), and using their mobile numbers obtained with permission, we located participants through multiple phone calls. When available, we collected demographic data for the non-responders including age, gender, and occupation.

### Data Collection

All data were collected using trained, local surveyors. Each participant completed a demographic and medical history survey which included a self-reported history of diabetes, hypertension, HIV, kidney disease, and heart disease (coronary, structural, or heart failure). If participants were receiving biomedical treatment in the form of medical therapy, then we collected specific drug information. Women additionally gave a self-reported history for pregnancy and menstruation. Awareness was defined as giving a self-reported disease history and subsequently testing positive in our screening process.

Anthropomorphic data (including height, weight, and body mass index) were recorded for each participant. Overweight was defined as a body mass index (BMI) greater than 25 kg/m^2^ and obesity was defined as a BMI greater than 30 kg/m^2^.

To detect CKD we obtained a serum creatinine and urine albumin. The serum creatinine was measured using the Cobas Integra 400 Plus (Roche Diagnostics; Basel Switzerland) at the Kilimanjaro Christian Research Institute Biotechnology Laboratory. The laboratory is known in the region for its high quality results, and it participates fully in international external quality assurance programs including the College of American Pathologists and the United Kingdom External Quality Assessment Service. All laboratory investigations were conducted according to Good Clinical Laboratory Practice standards. Urine albumin was detected with a Siemens MicroAlbustix (Siemens Healthcare Diagnostics, Inc.; Tarrytown, NY) from a mid-stream urine sample. All positive urine albumin results were confirmed with a repeat measurement from a first-morning void, mid-stream urine sample more than 48 hours from the initial measurement.

CKD was defined according to the Kidney Disease Improving Global Outcomes Working Group guidelines [18]. To estimate glomerular filtration rate (eGFR), we used the Modification of Diet in Renal Disease (MDRD) equation without the race factor which has been suggested to be the most accurate estimator of GFR in similar populations [19]. Stage I was defined as albuminuria with an eGFR greater than 90 ml/min/1.73m^2^, Stage II as albuminuria with an eGFR between 60 and 90 ml/min/1.73m^2^, Stage III as an eGFR between 30 and 60 ml/min/1.73m^2^ with or without albuminuria, Stage IV as an eGFR between 15 and 30 ml/min/1.73m^2^ with or without albuminuria, and Stage V as an eGFR less than 15 ml/min/1.73m^2^ with or without albuminuria. A urine albumin greater than 30 mg/dL in the absence of gross hematuria or an ongoing urinary tract infection as confirmed by urinalysis (Siemens Multistix 10G Urinalysis test strips) was considered positive. Quality control measures were performed weekly on each open and new bottle of urine dipsticks according to the manufacturer’s recommendation.

We measured blood pressure using the automated Omron HEM-712 sphygmomanometer (Omron Healthcare, Inc.; Bannockburn, IL) which has an adjustable cuff size. The machine was calibrated monthly during data collection. All participants were seated in an erect position with feet flat on the floor for a minimum of five minutes before measurements. Hypertension was defined as a single blood pressure measurement of greater than 160/100 mmHg, a two-time average measurement of greater than 140/90 mmHg, or current known use of anti-hypertensive medications.

Hemoglobin (Hb) A1c was determined from a fingerstick whole blood sample using the Bayer A1c Now+ point-of-care device (Bayer Healthcare LLC; Sunnyvale, CA). Quality control measures were performed weekly according to the manufacturer’s recommendation. Diabetes mellitus was considered present if the HbA1c level was greater than 7.0% or current known use of anti-hyperglycemic medications. Pre-diabetes was defined as an HbA1c greater than 6.0%.

We tested for the presence of HIV 1 and 2 according to the 2012 Tanzanian National HIV Rapid Testing and Treatment Guidelines which included the use of an HIV Counseling and Testing nurse [20]. HIV infection was defined as a positive Alere Determine HIV -1/2 assay (Alere Medical Co. Ltd; Waltham, MA) confirmed by a Uni-Gold HIV assay (Trinity Biotech Manufacturing Ltd; Wicklow, Ireland), a self-reported history, or the ongoing use of highly active anti-retroviral therapy.

### Data Analysis

Data were analyzed using STATAv.13 (STATA Corp., College Station, TX). The median and inter-quartile ranges (IQR) were reported for each continuous variable. Prevalence estimates were sample-balanced using age- and gender-weights based on the 2012 urban and rural district-level census data. All p-values are two-sided at a 0.05 significance level. Crude and adjusted prevalence risk ratios (PRR) were estimated using generalized linear models with a log link.

A secondary aim of the analysis was to estimate and contrast the prevalence of CKD by urban and rural status. As such, step-wise model specification was carried out by using substantive knowledge of factors associated with an urban environment that were also considered potential risk factors for CKD. Candidate models were then compared by calculation of the mean squared error (MSE) and Akaike Information Criterion (AIC) values. We used a Chi-squared test to compare differences between groups, and we used Taylor Series linearization to account for the design effect on variance due to cluster sampling. The intra-cluster correlation (ICC) coefficient for CKD was estimated from a one-way random intercepts model using the analysis of variance (ANOVA) estimator [21, 22].

## Results

Between January and June 2014, we enrolled 481 adults from 346 households including 123 men (25.6%) and 358 women (74.4%) with a median age of 45 years (IQR 35–59). Most participants were urban residents (n = 370; 77%), ethnically Chagga (n = 288; 59.9%), farmers or daily wage-earners (n = 199; 41.4%), and had a primary school education (n = 349; 72.6%)(**Table 1**). Men (p<0.001) and adults 18–39 years old (p = 0.001) were more likely to be non-responders. The proportion of participants with a secondary or post-secondary education (22.4%) was slightly higher than the expected regional average (14.6%)(p = 0.02), but there were no significant differences in occupation between the responders and non-responders (p = 0.64).

**Table 1:** Characteristics of the Survey Sample.

Variable (n, %)	Urban (n = 370)	Rural (n = 111)	Total (n = 481)
**Gender**			
Male	92 (24.9%)	31 (27.9%)	123 (25.6%)
Female	278 (75.1%)	80 (72.1%)	358 (74.4%)
**Age**			
18–39 years old	138 (37.3%)	34 (30.6%)	172 (35.8%)
40–59 years old	145 (39.2%)	46 (41.5%)	191 (39.7%)
60+ years old	87 (23.5%)	31 (27.9%)	118 (24.5%)
**Ethnicity**			
Chagga	230 (62.2%)	58 (52.3%)	288 (59.9%)
Pare	35 (9.5%)	31 (27.9%)	66 (13.7%
Sambaa	18 (4.9%)	9 (8.1%)	27 (5.6%)
Other^§^	87 (23.5%)	13 (11.7%)	100 (20.8%)
**Education**			
None	27 (7.3%)	4 (3.6%)	31 (6.4%)
Primary	253 (68.4%)	96 (86.5%)	349 (72.6%)
Secondary	64 (17.3%)	10 (9.0%)	74 (15.4%)
Post-Secondary	25 (7.0%)	1 (0.9%)	27 (5.6%)
**Occupation**			
Unemployed^#^	71 (19.2%)	3 (2.7%)	74 (15.4%)
Farmer/Wage Earner	114 (30.8%)	85 (76.6%)	199 (41.4%)
Small Business/Vendors	143 (38.6%)	15 (13.5%)	158 (32.8%)
Professional^†^	42 (11.4%)	8 (7.2%)	50 (10.4%)
**Self-Reported Medical History**			
Diabetes	53 (14.3%)	8 (7.2%)	61 (12.7%)
Hypertension	113 (30.6%)	21 (19.1%)	134 (28.0%)
Stroke	6 (1.6%)	2 (1.8%)	8 (1.7%)
Heart Disease^*^	17 (4.6%)	1 (0.9%)	18 (3.7%)
Tuberculosis	10 (2.7%)	0 (0%)	10 (2.1%)
Hepatitis	12 (3.2%)	2 (1.8%)	14 (2.9%)
Malaria	329 (88.9%)	98 (88.3%)	427 (88.8%)
Cancer	6 (1.6%)	0 (0%)	6 (1.3%)
COPD	8 (2.2%)	0 (0%)	8 (1.7%)
HIV/AIDS	20 (5.4%)	1 (0.9%)	21 (4.4%)
Kidney Disease	14 (3.8%)	0 (0%)	14 (2.9%)

§ Other tribal ethnicities represented in our groups include Luguru, Kilindi, Kurya, Mziguwa, Mnyisanzu, Rangi, Jita, Nyambo, and Kaguru

# Includes housewives and students

† Professional includes any salaried position (e.g. nurse, teacher, government employee, etc.) and retired persons

* Heart Disease includes coronary disease, heart failure, or structural diseases

**Fig 2:**
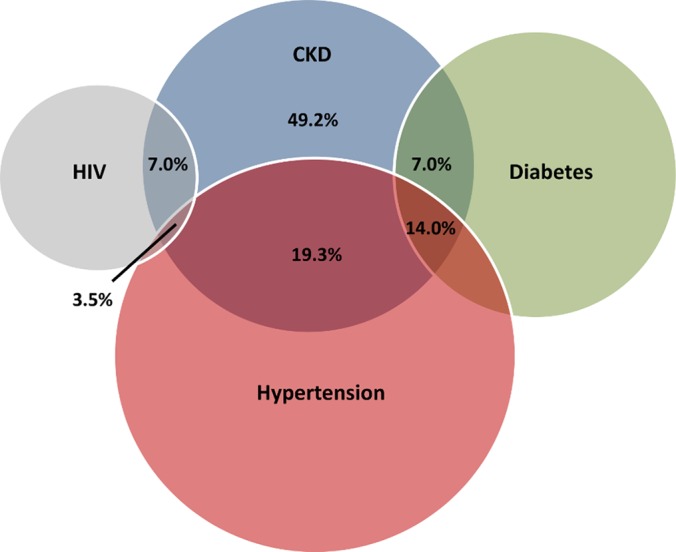
Venn diagram. The proportion of CKD associated with HIV, hypertension, and diabetes.

The overall prevalence of CKD was 7.0% (95% confidence interval [CI] 3.8–12.3). The design effect of the cluster-sampling for CKD was 1.80 with an ICC coefficient of 0.04. Most participants with CKD were classified as Stage I (43.8%) or Stage II (31.6%) with fewer participants having Stage III (21.1%), or Stage IV/V (3.5%). Among those with CKD, 7.0% had diabetes alone, 19.3% had hypertension alone, 14.0% had diabetes and hypertension, 7.0% had HIV alone, and 3.5% had HIV and hypertension. Half of the cases of CKD (49.2%) were not associated with any of the measured risk factors of hypertension, diabetes, or HIV (**Fig 2**).

We observed no difference in the prevalence of CKD by gender, age, ethnicity, education, tobacco use, or alcohol use (**Table 2**). Living in an urban setting (urbanicity), diabetes, HIV, a history of heart disease, and being employed as a professional were independently associated with higher prevalence risk ratios (PRR) for CKD. Participants with diabetes (PRR = 2.58; 95% CI 1.28–5.22) or HIV (PRR = 2.58; 1.01–6.57) had two and one-half-fold increased crude prevalence risk ratio for CKD, and participants with a history of heart disease (PRR = 3.03; 1.47–6.25) or who were employed as professionals (PRR = 3.41; 1.59–7.32) had more than a three-fold crude prevalence risk ratio for CKD. Participants with hypertension or who were unemployed or employed in a small-business/vendor tended to have higher prevalences of CKD, and although it was not at the 5% significance level, traditional medicine use had a strong association with CKD (PRR = 1.81; 0.90–3.63).

**Table 2:** Observed prevalence and model-predicted crude and adjusted prevalence risk ratios of CKD by demographic, health practices, socioeconomic, and co-morbid characteristics (CKD-AFRIKA 2014).

Variable	Chronic Kidney Disease		Prevalence Risk Ratios (95% CI)				
	Prevalence*	Cases (n/N)	Unadjusted	Adjusted			
	(95% CI)			Model 1	Model 2	Model 3	Model 4
**Demographics**							
** Age**							
18–39 years	7.6% (3.4–16.2)	16/172	1.00	1.00	1.00	1.00	1.00
40–59 years	5.4% (2.7–10.8)	24/191	1.35 (0.75–2.42)	1.38 (0.75–2.54)	1.35 (0.72–2.50)	1.31 (0.73–2.35)	1.35 (0.77–2.39)
60+ years	7.7% (3.3–16.6)	17/118	1.54 (0.78–3.08)	1.49 (0.69–3.24)	1.46 (0.70–3.05)	1.40 (0.65–2.99)	1.38 (0.66–2.90)
** Sex**							
Female	6.2% (3.6–10.3)	40/358	1.00	1.00	1.00	1.00	1.00
Male	7.9% (3.4–17.2)	17/123	1.23 (0.74–2.08)	0.78 (0.45–1.34)	0.75 (0.42–1.32)	0.69 (0.42–1.14)	0.67 (0.40–1.12)
** Ethnicity**							
Chagga	9.5% (4.7–18.3)	37/288	1.00	1.00	1.00	1.00	1.00
Pare	3.6% (0.6–18.2)	7/66	0.83 (0.34–2.03)	1.12 (0.60–2.08)	1.14 (0.57–2.27)	1.07 (0.51–2.24)	1.15 (0.54–2.43)
Sambaa	4.8% (0.5–33.7)	4/27	1.15 (0.40–3.33)	1.53 (0.63–3.71)	1.39 (0.58–3.34)	1.26 (0.51–3.11)	1.41(0.58–3.43)
Other	5.0% (1.5–14.7)	9/100	0.70 (0.36–1.36)	0.67 (0.33–1.35)	0.64 (0.32–1.27)	0.60 (0.30–1.22)	0.59 (0.29–1.17)
**Lifestyle Practices**							
** Ongoing Tobacco Use**	8.8% (2.5–26.9)	5/50	0.83(0.31–2.18)		0.71 (0.22–2.32)	0.68 (0.21–2.21)	0.77 (0.24–2.45)
** Ongoing Alcohol Use**	7.8% (3.4–16.7)	24/198	1.04 (0.61–1.77)		1.04 (0.60–1.80)	1.10 (0.66–1.83)	1.06 (0.63–1.77)
** Traditional Medicine Use**	8.0% (4.1–15.1)	40/272	1.81 (0.90–3.63)		1.83 (0.97–3.49)	1.81 (0.97–3.39)	1.78 (0.92–3.45)
**Socioeconomic**							
** Education**							
≤ Primary	6.5% (3.4–12.3)	44/380	1.00			1.00	1.00
≥ Secondary	8.8% (3.9–18.7)	13/101	1.11 (0.66–1.87)			0.76 (0.40–1.44)	0.75 (0.38–1.48)
** Occupation**							
Unemployed	9.2% (2.6–27.7)	10/74	1.92 (0.86–4.30)			1.57 (0.76–3.26)	1.54 (0.69–3.45)
Farmer/Wage Earner	4.7% (1.7–10.8)	14/199	1.00			1.00	1.00
Small-business/Vendor	10.1% (4.9–19.7)	21/158	1.89 (0.92–3.86)			1.63 (0.80–3.30)	1.49 (0.72–3.10)
Professional	16.7% (6.0–39.2)	12/50	**3.41 (1.59–7.32)**			**2.74 (1.31–5.75)**	**2.64 (1.19–5.86)**
**Co-Morbid Conditions**							
** Diabetes**	12.0% (4.7–27.3)	12/45	**2.58 (1.28–5.22)**				
** Hypertension**	5.2% (2.2–11.9)	21/149	1.30 (0.78–2.16)				
** Obesity**	4.7% (1.7–13.2)	14/138	0.81 (0.45–1.47)				
** History of Heart Disease**	49.6% (14.8–84.8)	6/18	**3.03 (1.47–6.25)**				
** Any NCD^†^**	5.40% (2.5–11.3)	34/259	1.27 (0.78–2.06)				1.21 (0.67–2.18)
** HIV**	13.7% (3.0–45.3)	6/21	**2.58 (1.01–6.57)**				**2.60 (1.12–6.07)**
**Urbanicity**							
** Rural**	2.0% (0.5–6.9)	3/111	1.00	1.00	1.00	1.00	1.00
** Urban**	15.2% (9.6–23.3)	54/370	**5.40 (2.05–14.2)**	**6.06 (2.15–17.1)**	**6.10 (2.27–16.4)**	**4.88 (1.75–13.5)**	**4.80 (1.70–13.6)**

*Gender- and age-weighted

† Includes a diagnosis of diabetes, hypertension, or obesity or a history of heart disease

**BOLD:** Significant at the 5% level

Urbanicity had the strongest association with CKD of any measured variable. We observed 54 cases of CKD in the urban setting and three cases in the rural setting, and compared to rural residents, urban residents had a five-fold higher crude prevalence risk ratio of CKD (PRR 5.40; 2.05–14.2). When we adjusted for other measured variables including demographics, lifestyle practices, socioeconomic indicators, and co-morbid conditions, the high prevalence risk ratio for urbanicity and CKD persisted (**Table 2**).

**Fig 3:**
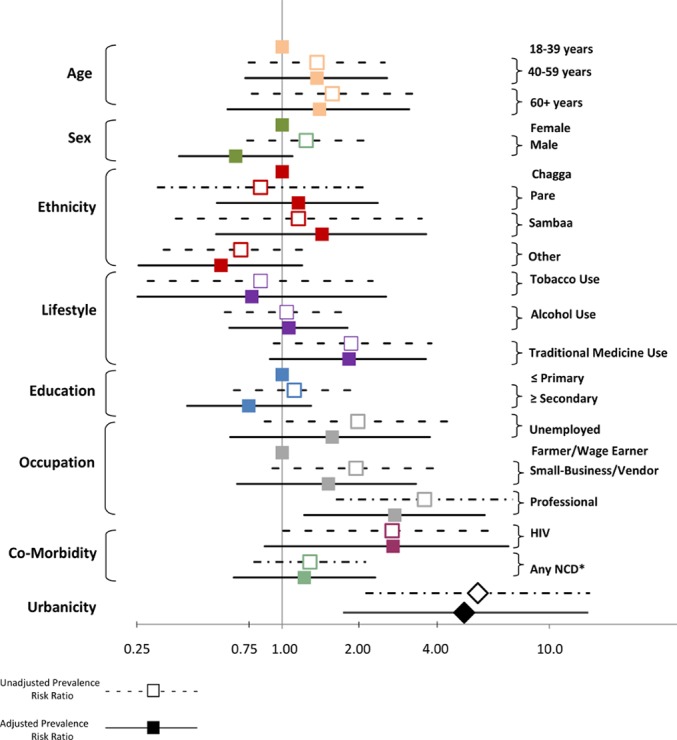
Forest plot. The crude and fully-adjusted (model 4) prevalence risk ratios for CKD by each variable relative to the reference group for each variable.

In the demographic-adjusted model (model 1), the prevalence risk ratio for CKD among urban residents was 6.06 (2.15–17.1). The addition of lifestyle practices to the adjusted-model (model 2) had little effect on the PRR for urbanicity while socioeconomic indicators and the presence of a co-morbid condition (diabetes, hypertension, HIV, obesity, or history of heart disease) accounted for 21% of the excess urban risk. In the fully-adjusted model (model 4), the prevalence risk ratio for CKD among urban residents was 4.80 (1.70–13.6), and the attributable prevalence risk of CKD for the urban population by unmeasured factors was 79%. The strong relationship between professional occupation (PRR = 2.64; 1.19–5.86) and HIV (PRR = 2.60; 0.66–2.18) and CKD remained in the fully-adjusted model (**Fig 3**). In this model, older participants tended to have higher prevalence estimates, and traditional medicine use also retained a strong association with CKD (PRR = 1.78; 0.92–3.45).

Awareness of CKD was 10.5% (4.7–22.0) among participants found to have CKD. Awareness did not vary significantly by age, gender, ethnicity, education, urbanicity or stage of CKD. Among those with concurrent co-morbidities including CKD and hypertension or CKD and diabetes, 28% (13.2–49.0) and 11.7% (2.5–40.8) respectively were receiving any biomedical treatment for their diabetes or hypertension.

## Discussion

Among a randomly-selected, community-based sample in Northern Tanzania, we observed a high prevalence of CKD in the context of concomitant NCDs; however, NCDs accounted for only a modest proportion of the significantly higher prevalence of CKD among urban residents compared to their rural counterparts. Despite this high urban burden of CKD that is comparable to high- and middle-incomes countries, CKD awareness was low, and few persons were receiving any biomedical therapy for co-morbidities that could slow CKD progression [23, 24].

In high-income countries, urban risk factors include lifestyle changes and a higher presence of other chronic conditions such as diabetes and hypertension, but our results demonstrated that these traditional urban risk factors alone did not explain the excess risk of CKD experienced by urban residents in Northern Tanzania. In our study, the majority of the attributable prevalence risk for urbanicity and CKD remained unexplained after adjusting for other measured co-variables including age, gender, ethnicity, education, occupation, alcohol use, tobacco use, traditional medicine use, NCDs, and HIV. This may be related to the lack of any associated potential etiologies in half the CKD cases we identified, and it is consistent with studies from LMICs in other regions showing high urban rates of CKD that are only partly attributable to NCDs like diabetes or hypertension [25–27].

In sub-Saharan Africa, rapid urbanization has led to densely crowded cities with unplanned infrastructure, poor sanitation and waste disposal, and heavy environmental pollutions [28]. As a result, ongoing exposure to environmental toxins and infectious diseases exist alongside increasing rates of NCDs such as hypertension and diabetes [29]. Our study findings, which suggested that these NCDs accounted for only part of the increased urban prevalence of CKD, emphasizes the vulnerability of the people in East Africa to CKD which may be caused by exposure to environmental toxins in addition to communicable and non-communicable diseases. To better understand the specific etiologies causing CKD, larger-scale epidemiological studies are needed to examine many potential but currently unmeasured urban risk factors such as contaminated water supplies, occupational exposures, unregulated food additives, increased access to over-the-counter analgesics, and different infectious diseases [30].

To our knowledge, this is the first randomly-sampled, community-based household-level survey that has assessed for CKD in East Africa. Moreover, we examined CKD in the context of multiple risk factors including hypertension, diabetes, and HIV which are especially prevalent in East Africa [12–15]. In addition, these results are externally generalizable because the population of the Kilimanjaro Region is comparable to the national population of Tanzania [5]. Other strengths of our study include the use of clinically meaningful disease indicators, the use of standardized definitions of disease including confirmation of urine albumin by repeat assessment, and the use of a high quality laboratory for serum creatinine measurements.

Despite these strengths, there are also several limitations of our study. Given the cross-sectional design, causal relationships between measured risk factors and CKD cannot be drawn, and some of the measured variables included in our models are only partially representative of the underlying constructs, e.g. we used education and occupation as proxies for socioeconomic status because we did not measure household income. Further, the strongest model-estimated association was between urbanicity and CKD, and although this association was robust to adjustment for other explanatory variables, it was based on few cases in the rural setting. We did not repeat urine albumin measurements on participants who initially screened negative which may have led to an underestimate of CKD in our study, and even though we observed high rates of early stage CKD, it is not clear to what extent survivor bias affected this. We also only obtained serum creatinine measurements at a single time point, so it is possible that acute kidney injury was misclassified as CKD in some instances. Additionally, we did not collect a dietary history (e.g. meat intake) from participants which could have led to unrecognized variance in creatinine values among participants. Misclassification of disease around the cutoff points for hypertension and diabetes may also be present although we expect this misclassification to be non-differential. Non-response bias can be introduced when the response rate is low and when there is a substantial difference between responders and non-responders [31]. To address any non-response bias that may have arisen from differences between the respondents and non-respondents, we used sample-balanced weights for age and gender, and we explored differences in occupation and education level between the two groups.

In conclusion, NCDs are a growing global health burden that is increasing rapidly in LMICs where they overlap with high rates of infectious diseases and environmental toxic exposures. As demographic transitions reshape sub-Saharan Africa, countries like Tanzania are at risk for an explosive growth in the burden of CKD. In order for the morbidity and mortality from NCDs to be effectively reduced, CKD must be included in broad public health efforts that already target identification of the major etiologies of CKD. Our findings demonstrated a high prevalence of CKD in a sub-Saharan African country, and they provide some of the strongest evidence to date for the importance of including CKD in the widespread efforts to address NCDs.

## Supporting Information

S1 Appendix Standard Operating Protocol (SOP) for Household Selection (DOCX)
